# Weekly epirubicin in patients with hormone-resistant prostate cancer

**DOI:** 10.1038/sj.bjc.6600525

**Published:** 2002-09-23

**Authors:** R Petrioli, A I Fiaschi, D Pozzessere, S Messinese, M Sabatino, S Marsili, P Correale, A Manganelli, F Salvestrini, G Francini

**Affiliations:** Medical Oncology Division, Institute of Internal Medicine, University of Siena, Siena, Italy; Pharmacology Department, University of Siena, Siena, Italy; Urology Department, University of Siena, Siena, Italy; Clinical Urology, University of Siena, Siena, Italy

**Keywords:** hormone-refractory prostate cancer, androgen-independent prostate cancer, bone metastasis, quality of life

## Abstract

The aim of this study was to investigate the benefit of weekly epirubicin in the treatment of metastatic hormone-resistant prostate cancer. One hundred and forty-eight patients with metastatic hormone-resistant prostate cancer received weekly 30-min intravenous infusions of epirubicin 30 mg m^2^ of body surface area. The primary end-point was palliative response, defined as a reduction in pain intensity and an improvement in performance status. The secondary end-points were the duration of the palliative response, quality of life and survival. Fifty-seven (44%) of the 131 evaluable patients met the primary criterion of palliative response after six treatment cycles and 73 (56%) after 12 cycles; the median duration of the response was 9 months (range 1–11). The median global quality of life improved in 52% of the patients after six cycles and in 68% after 12 cycles. The 12- and 18-month survival rates were respectively 56 and 31%, with a median survival of 13+ months (range 1–36). The treatment was well tolerated: grade 3 neutropenia was observed in 8% of the patients, grade 3 anaemia in 7%, and grade 3 thrombocytopenia in 3%. None of the patients developed grade 4 toxicity or congestive heart failure. Weekly epirubicin chemotherapy can lead to a rapid and lasting palliative result in patients with metastatic HRPC, and have a positive effect on the quality of life and survival.

*British Journal of Cancer* (2002) **87**, 720–725. doi:10.1038/sj.bjc.6600525 www.bjcancer.com

© 2002 Cancer Research UK

## 

Prostate carcinoma remains the most common cancer in males. Both surgery and radiation therapy are potentially curative provided that the tumour is confined to the prostate itself but, if the lymph nodes are involved, the possibility of dissemination is high. Hormonal therapy remains a standard and effective systemic approach to disseminated disease (Catalona, 1994), but it is unfortunately successful in only 70–80% of patients and the median duration of response is no longer than 12–24 months (Gittes, 1991). The median survival of men with hormone refractory disease has increased over the last few years from 9–12 to 15–20 months, more as a result of the earlier detection of androgen-insensitive disease by means of PSA levels rather than symptoms (Daneshgari and Crawford, 1993; Hussain *et al*, 1994; Knox and Moore, 2001).

The role of chemotherapy in the treatment of hormone-resistant prostate carcinoma (HRPC) has not yet been established. Most previous studies have found little evidence of anti-tumour activity, with response rates of less than 10% for single chemotherapeutic agents and similar results for combination therapies (Eisenberger *et al*, 1985; Millikan, 1999). However, more recent studies with mitoxantrone, docetaxel and estramustine have shown a response (Tannock *et al*, 1996; Savarese *et al*, 2001). It is difficult to assess the effectiveness of chemotherapeutic agents in the treatment of metastatic prostate cancer because the patients often do not have measurable disease (Coleman, 1998). One of the biggest problems is monitoring the response in bone, which is the only site of metastases in most patients. Various tumour and bone turnover markers can be useful in assessing true changes in bone lesions and, in this setting, one of our recent studies showed that urinary calcium excretion was the most useful marker for monitoring clinical response (Francini *et al*, 2001).

Doxorubicin has shown some activity in the treatment of HRPC patients (Torti *et al*, 1983; Scher *et al*, 1984), and we have previously reported the satisfactory effectiveness of the doxorubicin analogue epirubicin which, at equipotent doses, is associated with quantitatively less severe toxicities than its parent compound (Francini *et al*, 1993). However, our results were based on standard National Prostatic Cancer Project (NPCP) criteria that no longer seem to be satisfactory for evaluating response (Slack and Murphy, 1984).

The present extended study coordinated by the University of Siena's Oncopharmacology Centre was performed in order to verify the palliative response of epirubicin-based chemotherapy on the basis of validated criteria such as pain and performance status.

## PATIENTS AND METHODS

### Eligibility criteria

The study involved patients with histologically confirmed measurable or evaluable metastatic prostatic carcinoma that had progressed during hormonal therapy. The patients were admitted to the chemotherapy protocol provided that they met at least one of the following criteria: an increase in PSA level of ⩾50% in comparison with baseline on two successive occasions separated by at least 2 weeks; new metastatic lesions revealed by a bone scan; an increase of more than 25% in a bidimensionally measurable tumour mass. The patients treated with LH-RH agonists had to continue their primary androgen ablation therapy, and were required to have serum testosterone levels <30 ng ml^−1^ before study entry. Anti-androgens had to be stopped 4–6 weeks before the use of chemotherapy in order to allow the withdrawal to become effective. All of the patients had symptoms that included pain. All of the patients had to have a Karnofsky performance status (KPS) of ⩾50, and adequate haematological (leukocytes ⩾3000 μl^−1^; haemoglobin ⩾8 g dL^−1^, platelets ⩾100 000 μl^−1^), renal (serum creatinine ⩽2.0 mg dl^−1^), and hepatic function (serum bilirubin ⩽2.0 mg dl^−1^). Patients with widespread bone metastases or platelet counts of between 50 000 and 100 000 μl^−1^ were also included in the study. The exclusion criteria were previous chemotherapy, congestive heart failure, a recent myocardial infarction, or other previous malignant diseases except basal cell carcinoma or squamous cell carcinoma of the skin. All of the patients gave their informed consent, and the protocol was approved by the Ethics Committee of the Siena University.

### Assessment of response and quality of life

The pretreatment evaluation included a complete physical examination and the determination of a baseline KPS score. The primary end-point was a palliative response, which was defined as a 2-point reduction in the 6-point present pain intensity scale of the McGill-Melzack Pain Questionnaire (or the complete disappearance of pain if the initial score was 1+), and an improvement in KPS of one 10-point category from baseline (Melzack, 1975). These results had to be maintained at two consecutive evaluations made at least 3 weeks apart and without any increase in analgesic consumption. The pain scale has verbal descriptors (0=no pain, 1=mild pain, 2=discomforting pain, 3=distressing pain, 4=horrible pain, and 5=excruciating pain), and the patients were asked to classify the average pain level during the previous 24 h. We used a translated form of the McGill Melzack Questionnaire to which the ‘reconstruction-based methodology’ has been applied (de Benedittis *et al*, 1988).

The other end-points of the study were the duration of the palliative response (as defined by the primary end-point), a >50% decrease in PSA levels compared with baseline on two successive occasions separated by a period of at least 2 weeks, quality of life and survival. Progression was defined as an increase in the present pain intensity scale of >1 point above the nadir or a >25% increase in analgesic consumption in comparison with baseline, each recorded at two consecutive visits; unequivocal evidence of new lesions, radiological progression, or the need for radiation therapy also constituted disease progression. Analgesic consumption was based on the average daily quantities taken by the patient during the previous week.

PSA progression was defined as two consecutive increases in serum PSA concentrations to more than 50% above baseline or nadir levels. The patients with stable serum PSA concentrations included those without PSA progression but with a less than 50% decrease in baseline levels.

In order to assess the effects of disease and treatment on the patients' health-related quality of life, they were asked to complete the European Organization for Research and Treatment of Cancer (EORTC) core questionnaire (EORTC/QLQ-C30) at trial entry and then every 3 weeks. This questionnaire, which has been translated and validated in 38 languages including Italian, consists of 30 ordinal scale items that include multi-item domains for physical function, emotional function, social function, pain, and the global quality of life, and individual items including fatigue, appetite and constipation (Aaronson *et al*, 1993).

The laboratory studies included blood and platelet counts and a comprehensive screening profile (alkaline phosphatase, blood urea nitrogen, creatinine, calcium, phosphorus, uric acid, total protein, albumin, total bilirubin and electrolyte levels) at baseline and every 3 weeks. Serum PSA, serum calcium, the urinary calcium/creatinine ratio (UCa/Cr), serum phosphate, the urinary phosphate/creatinine ratio, serum bone alkaline phosphatase, serum procollagen type I carboxy-terminal pro-peptide, the urinary hydroxyproline/creatinine ratio, and serum carboxy-terminal telopeptide of collagen type I were all measured using standard methods at baseline and after six and 12 chemotherapy cycles (Francini *et al*, 1988, 1992). The patients underwent a weekly complete blood cell count before chemotherapy. The imaging studies included an abdominal and pelvic CT scan or magnetic resonance imaging, a bone scan and chest radiograph. All measurable disease was re-evaluated at 12-week intervals, and radionuclide bone scans were repeated every 12 weeks. In all cases, a baseline ECG was obtained, and a further cardiac work-up was performed if indicated. The palliative response and quality of life data were reviewed by an independent external consultant.

### Treatment plan

Treatment consisted of 30-min intravenous infusions of epirubicin 30 mg m^2^ of body-surface area, which were given at 1-week intervals if serum WBC levels were >3000 μl^−1^, granulocytes >1500 μl^−1^, and platelets >100 000 μl^−1^. The platelet counts of the patients enrolled with levels of between 50 000 and 100 000 μl^−1^ had to normalise during the first three chemotherapy cycles; if not, the treatment was discontinued. Chemotherapy was given until progression or the onset of severe toxicity. In order to minimise the probability of cardiac toxicity, it was recommended that the patients still responding after a cumulative epirubicin dose of 720 mg m^−2^ continue treatment with the best supportive care alone. Metoclopramide was used as antiemetic medication; dexamethasone or other steroids were not used.

The patients continued to take analgesic medication at doses adjusted in order to provide optimal pain control. Bisphosphonates were not used. The chemotherapy was initally administered in hospital to the patients whose KPS was between 60 and 50%; if their performance status improved during treatment, it was subsequently administered on an outpatient basis.

### Treatment-related adverse events

These were assessed weekly using World Health Organisation (WHO) criteria (Miller *et al*, 1981). The treatment was interrupted at the first occurrence of grade II toxicity and resumed at the same dose after resolution to grade I or better, with prophylaxis being given when possible. In the case of grade III or IV toxicity, the treatment was interrupted and a maximum of 3 weeks were allowed for recovery, after which the patients were withdrawn from the study. Anaemia was treated as clinically indicated; nausea and vomiting were treated symptomatically. Haematopoietic growth factors were not routinely used but given only when neutrophil counts fell below 1000 m^3^ and continued until complete haematological recovery.

### Statistics

In accordance with Simon's two-stage phase II design, a sample size of 110 patients was required, assuming a palliative result of approximately 30% for other treatment modalities and a target activity level of interest of 45%, with an α of 0.05 and a β of 0.90. It was planned to enrol a further 20 patients in the expectation of at least 10–15% of unevaluable cases.

Differences in the QL measures between baseline and the subsequent treatment cycles were assessed using a paired *t-*test. The duration of survival was measured from the date of first treatment to the date of death or last follow-up.

## RESULTS

Between February 1996 and June 2001, 148 patients with HRPC were enrolled; their median age was 67 years (range 49–75). The initial KPS was 90% in 27 patients, between 80% and 70% in 96, and between 60% and 50% in 25. One hundred and forty-two patients had bone metastases; the other six (4%) had other than bone metastases and measurable disease (Table 1

**Table 1:**
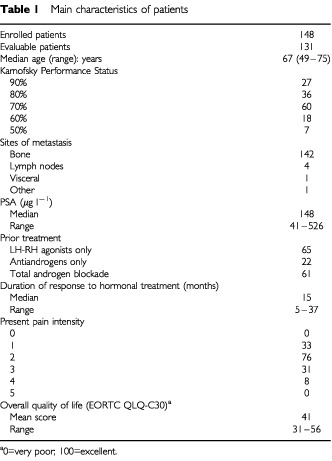
Main characteristics of patients

).

Palliative response was evaluable in 131 patients (seven patients received only one or two treatment cycles, three refused to continue after two treatment cycles, two died of causes other than cancer before the fourth treatment cycle, and five were excluded because of corticosteroid use). The 131 evaluable patients received a total of 1765 cycles (median per patient 13 cycles; range 3–24). A palliative response was observed in 57 patients (44%) after six treatment cycles and 73 (56%) after 12 cycles (Table 2

**Table 2:**
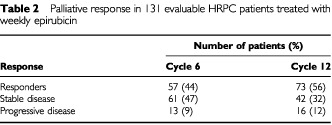
Palliative response in 131 evaluable HRPC patients treated with weekly epirubicin

). The median response duration was 9 months (range 1–11). Most of the patients experienced a rapid and progressive decrease in pain intensity and analgesic medication during the first three treatment cycles, as well as an improvement in KPS. After 12 treatment cycles, pain intensity had stabilised in 42 patients without any increase in analgesic consumption for at least 3 weeks after the beginning of chemotherapy, whereas 16 patients experienced progressive disease as shown by worsening pain or KPS and/or new bone lesions. The disease stabilised in four of the six patients with measurable lesions, and progressed in the remaining two. On the basis of the change in PSA levels, 32 (24%) of the 131 evaluable patients showed a PSA response, 54 (41%) stable disease, and 45 (35%) progression. Among the 73 patients who achieved a palliative response after 12 treatment cycles, UCa/Cr increased by >50% in 27 (37%) and PSA decreased by >50% in 22 (30%) (*P*=0.4; χ^2^-test); among the 58 non-responders, UCa/Cr increased by >50% in only two (3%) and PSA decreased by >50% in 10 (17%) (*P*=0.03; χ^2^-test) (Figure 1

**Figure 1:**
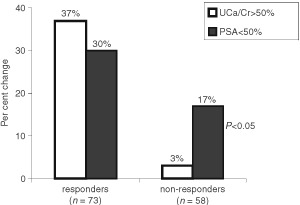
Per cent change in UCa/Cr and PSA in responders and non-responders, after 12 treatment cycles. *P*<0.05 (X^2^ -test), significantly different between PSA and UCa/Cr in non-responding patients; in parentheses, *n*=number of patients.

).

### Quality of life and survival

**Figure 2:**
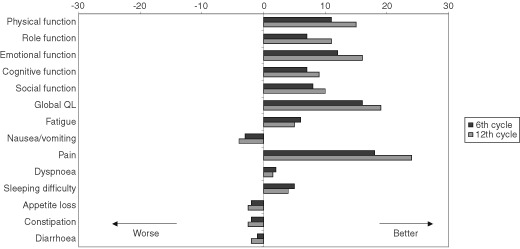
Mean changes in QLQ-C30 scores.

**Figure 3:**
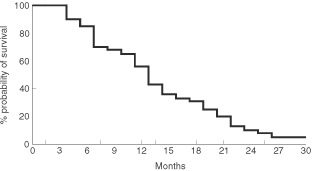
Overall survival of 148 HRPC patients treated with weekly epirubicin.

Baseline QLQ-C30 data were available for 127 patients (a compliance rate of 86%); the remaining patients were excluded from the further serial analyses. For ease of interpretation, all of the scale and item scores were linearly transformed to a 0–100 scale. The baseline scores suggested that the patients were highly symptomatic. Compliance with QL assessment decreased to 94 patients (63%) after six treatment cycles and 77 (52%) after 12 cycles. The main reasons for non-compliance were disease progression, unacceptably completed forms and/or death. Figure 2

shows the mean changes in the scores from baseline to cycles 6 and 12. By cycle 12, there were significant differences from baseline in physical and emotional function, global QL and pain scores (*P*<0.01, *P*<0.01, *P*<0.005, *P*<0.001, respectively). The mean global QL improved in 52% of the patients after six treatment cycles and in 68% after 12 cycles. A total of 128 patients had died as of December 2001. The survival rate was 56% after 12 months and 31% after 18 months, with a median survival of 13+ months (range 1–36) (Figure 3

).

### Toxicity

The chemotherapy was well tolerated and there were no treatment-related deaths. The majority of patients continued to work full time, with treatment having a minimal impact on normal daily activities. Neutropenia and anaemia were the most frequent side effects, but were grade I or II in most cases; grade III neutropenia occurred in 8% of the patients and grade III anaemia in 7%. Grade III thrombocytopenia occurred in 3% of the patients. None of the patients experienced grade IV toxicity. The administration of at least one treatment cycle was delayed by 1 week in 36 patients: the reasons for the delays were haematological in 29 cases (22%) and non-haematological (stomatitis, nausea) in seven (5%). Sixteen patients showed an improvement in anaemia and platelet counts after 1–3 treatment cycles. Twenty-four patients, who received cumulative doses of 720 mg m^2^, did not develop clinical congestive heart failure, but two developed atrial fibrillation that was medically controlled without further cardiac complications. Performance status also improved in many of the patients with an initial KPS of between 60 and 50%, so that the subsequent cycles could be administered on an outpatient basis; a total of 93% of all of the chemotherapy cycles were given in this manner.

## DISCUSSION

Most cytotoxic drugs, alone or in combination, have usually led to low response rates and a brief median survival in patients with HRPC. More recently, taxanes and new drug combinations have demonstrated some activity in this disease, partially due to a reconsideration of end points such as QL parameters (Tannock *et al*, 1996; Picus and Schultz, 1999; Kelly *et al*, 2001).

The results of this study provide evidence of the palliative effect of weekly epirubicin chemotherapy in 131 evaluable patients with HRPC. A palliative response (defined as an improvement in the pain index and performance status) was observed in 44% of the patients after six treatment cycles, and 56% after 12 cycles (Table 2). This high response rate may be related to the real anti-tumour activity of epirubicin, and/or the baseline characteristics of the patients. It is known that some prognostic factors, such as a long period of responsiveness to previous hormonal therapy, a good performance status and a limited extension of bone metastases are usually correlated with a more favourable disease outcome (Fossa *et al*, 1992; Hussain *et al*, 1994). About 85% of our patient population had responded to previous hormonal treatment for >12 months, but most of them had widespread bone metastases and a poor performance status. A detailed statistical study of prognostic factors in HRPC will be presented in the future.

Our results correlated with changes in bone and tumoral markers: the concordance of an increase in UCa/Cr in responders was not significantly different from the decrease in PSA, whereas the concordance of these two indices in non-responders was statistically significant (Figure 1). This finding suggests that the change in UCa/Cr agrees better with a palliative response than the change in PSA. The correlation between the changes in UCa/Cr and response or non-response is supported by one of our previous studies showing that UCa/Cr is the best marker for monitoring bone metastases from prostate cancer (Francini *et al*, 2001).

The median duration of the palliative response was about 10 months, which is similar to that recently observed with the use of mitoxantrone + prednisone or taxotere, and longer than that found with prednisone alone, vinorelbine or paclitaxel (Tannock *et al*, 1996; Fields-Jones *et al*, 1999; Trivedi *et al*, 2000; Savarese *et al*, 2001). The palliative response was accompanied by an improvement in a number of the quality of life dimensions, particularly a reduction in pain and an improvement in functional scales. It has been shown that pain and reduced physical function are the most frequent complaints in HRPC patients, and so improving these dimensions represents an important treatment goal (Moore *et al*, 1994). Moreover, the improvement in performance status during treatment in many patients with an initial KPS of between 60 and 50% led to them being treated on an outpatient basis after 2–6 cycles received as in-patients. However, given that compliance with QL data assessment was low, it is difficult to draw strong conclusions from the QL data.

The proposed weekly epirubicin chemotherapy was well tolerated and did not cause any unexpected toxicities. The main toxicity was mild myelosuppression, but decreased haemoglobin and platelet levels may reflect the metastatic invasion of bone marrow or uremic marrow suppression, and sometimes urinary blood losses (Berry *et al*, 1979). It is worth noting that 16 patients showed an improvement in bone marrow reserves, with increased haemoglobin levels and platelet counts after 1–3 treatment cycles, a finding which suggests that epirubicin is active against the neoplastic invasion of bone marrow from prostate cancer. No case of congestive heart failure was observed probably because weekly anthracycline schedules are less cardiotoxic than 3-weekly administrations, and are well tolerated even in elderly populations such as that represented by prostate cancer patients (Chlebowski *et al*, 1980). Another factor contributing to the absence of severe cardiotoxicity was the planned discontinuation of chemotherapy at the cumulative dose of 720 mg m^−2^. In order to increase efficacy, higher doses of epirubicin (100 mg m^−2^ i.v. every 3 weeks) have been tested, with a 24% partial response rate based on WHO criteria (Brausi *et al*, 1995). Interesting results have been reported with epirubicin 100 mg m^−2^ every 3 weeks plus estramustine phosphate (54% of the patients achieved a ⩾50% reduction in PSA); however, cardiovascular toxicity requiring hospitalisation and grade 3–4 myelotoxicity occurred (Hernes *et al*, 1997). Our results suggest that reducing the individual doses of epirubicin may be preferable in clinical use and allow prolonged periods of treatment in HRPC.

The median survival of our study population (13+ months) was slightly better than that found in most series (8–10 months) treated with other cytotoxic drugs or placebo (Tannock *et al*, 1996; Heidenreich *et al*, 2001; Kelly *et al*, 2001). Although ours was not a randomised trial, it is possible that an active and safe chemotherapy such as weekly epirubicin may prolong survival with a relatively good quality of life in HRPC patients.

In conclusion, weekly epirubicin chemotherapy can lead to a rapid and lasting palliative result, and have a positive effect on the quality of life and duration of survival, in patients with metastatic HRPC. It is also worth noting that, even in this advanced disease stage, weekly epirubicin could be administered on an outpatient basis with mild toxicity.
